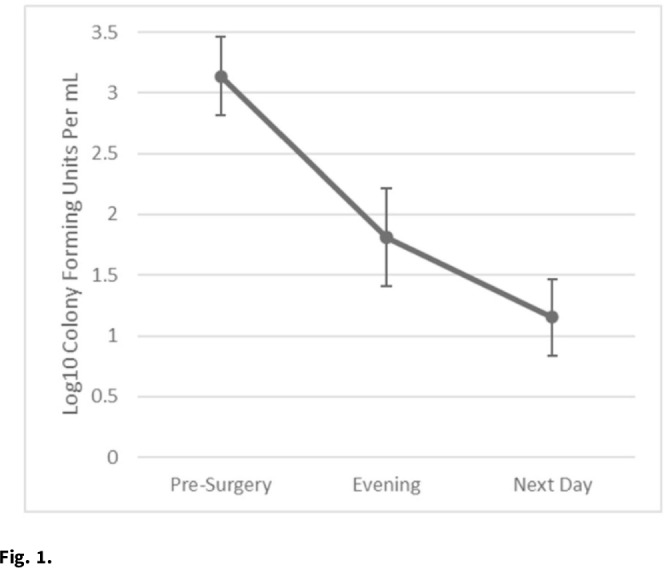# Feasibility and acceptability of intranasal povidone iodine decolonization among orthopedic trauma surgery patients

**DOI:** 10.1017/ash.2022.177

**Published:** 2022-05-16

**Authors:** Marin Schweizer, Loreen Herwaldt, Linda Boyken, Jean Pottinger, Rachel Quinn, Daniel Diekema, Fiona Armstrong Pavlik, Melissa Ward, Poorani Sekar, Michael Willey

## Abstract

**Background:** Nasal decolonization significantly decreases the incidence of *Staphylococcus aureus* surgical-site infections (SSIs). Patient adherence with self-administration of a decolonization ointment (ie, mupirocin) is low, especially among patients having urgent surgery. Povidone-iodine decolonization may overcome patient adherence challenges because povidone-iodine needs to be applied only on the day of surgery. We assessed the effectiveness and acceptability of povidone-iodine decolonization given on the day of surgery among patients having orthopedic trauma surgery. **Methods:** Adult patients who underwent operative fixation of traumatic lower extremity fractures were consented to receive 10% intranasal povidone-iodine solution. Povidone-iodine was applied ~1 hour before surgical incision and was reapplied the evening after surgery. Patients were tested for *S. aureus* nasal colonization before surgery, the evening after surgery (before povidone-iodine reapplication), and the day after surgery. Swabs were inoculated into Dey-Engley neutralizer and processed in a vortexer. A series of dilutions were performed and plated on mannitol salt agar plates. *S. aureus* cultures were quantitatively assessed to determine the reduction in *S. aureus* after povidone-iodine use. Reductions in *S. aureus* nasal growth were evaluated using the Skillings-Mack test. SSIs manifesting within 30 and 90 days of surgery were identified using NHSN definitions. A survey was administered the morning after surgery to determine the acceptability of intranasal povidone-iodine. **Results:** In total, 51 patients participated in this pilot study between February 2020 and June 2021. Nasal samples from 12 participants (23.5%) grew *S. aureus*. The *S. aureus* concentration decreased significantly across the time points (*P* = .03) (Fig. 1). No SSIs were identified within 30 days of surgery. One SSI occurred within 90 days of surgery; this patient did not carry *S. aureus*, and cultures from the infected site were negative. Also, 31% of patients reported at least 1 mild side effect while using povidone-iodine: dripping (n = 7), itching (n = 6), dryness (n = 4), stinging (n = 4), staining (n = 3), unpleasant taste (n = 3), runny nose (n = 2), burning (n = 1), sneezing (n = 1), sore throat (n = 1), tickling (n = 1), and/or cough (n = 1). Also, 86% of patients stated that povidone-iodine felt neutral, pleasant, or very pleasant, and only 14% stated that it felt unpleasant or very unpleasant. **Discussion:** In this pilot study, 2 applications of nasal povidone-iodine on the day of surgery were acceptable for patients, and this protocol significantly reduced *S. aureus* concentration in nares of patients. Future large clinical trials should evaluate whether this 2-application regimen of povidone-iodine significantly decreases rates of SSI among orthopedic trauma surgery patients.

**Funding:** PDI Healthcare

**Disclosures:** None

**Figure:**